# Occurrence of Central Nervous System Complications of Respiratory Syncytial Virus Infections: A Systematic Review with Meta-Analysis

**DOI:** 10.3390/epidemiologia5030031

**Published:** 2024-07-19

**Authors:** Matteo Riccò, Antonio Cascio, Silvia Corrado, Marco Bottazzoli, Federico Marchesi, Renata Gili, Pasquale Gianluca Giuri, Davide Gori, Paolo Manzoni

**Affiliations:** 1 AUSL–IRCCS di Reggio Emilia, Servizio di Prevenzione e Sicurezza Negli Ambienti di Lavoro (SPSAL), Local Health Unit of Reggio Emilia, 42122 Reggio Emilia, Italy; 2 Infectious and Tropical Diseases Unit, Department of Health Promotion, Mother and Child Care, Internal Medicine and Medical Specialties, “G D’Alessandro”, University of Palermo, AOUP P. Giaccone, 90127 Palermo, Italy; antonio.cascio03@unipa.it; 3 ASST Rhodense, Dipartimento della donna e Area Materno-Infantile, UOC Pediatria, 20024 Milan, Italy; 4 Department of Otorhinolaryngology, APSS Trento, 38122 Trento, Italy; 5 Department of Medicine and Surgery, University of Parma, 43126 Parma, Italy; 6 Department of Prevention, Turin Local Health Authority, 10125 Torino, Italy; 7 Department of Medicine and Diagnostics, AUSL di Parma, 43100 Parma, Italy; 8 Department of Biomedical and Neuromotor Sciences, University of Bologna, 40126 Bologna, Italy; 9 Department of Public Health and Pediatric Sciences, University of Torino School of Medicine, 10125 Turin, Italy

**Keywords:** respiratory syncytial virus, respiratory tract infections, seasonal influenza, encephalitis, epidemiology

## Abstract

An increasing base of evidence suggests that respiratory syncytial virus (RSV) infections may be associated with neurological complications. In accord with the PRISMA statement, we performed a systematic review and meta-analysis on the occurrence of encephalitis and encephalopathy associated with documented RSV infections. PubMed, Embase, and Scopus databases were searched for eligible observational studies published up to 10 April 2024. Raw data included the occurrence of RSV infections among cases of encephalitis and/or encephalopathy and cases of encephalitis and/or encephalopathy among series of RSV infections. Data were pooled in a random effects model. Case reports were also collected, and their data pooled as a cumulative series. Heterogeneity was assessed using the I^2^ measure, while reporting bias was assessed by means of funnel plots and regression analysis. A total of 15 studies for a total of 7719 RSV infections and 1631 cases of encephalitis were analyzed. Moreover, 27 case reports and case series were retrieved, for a total of 84 individual cases of encephalitis/encephalopathy occurring during a documented RSV infection. A pooled prevalence of 2.20 cases of encephalitis/encephalopathy per 100 RSV cases (I^2^ = 99%) was calculated, while a prevalence of RSV infections among cases of encephalitis/encephalopathy was estimated to 3.53 per 100 cases for studies on respiratory specimens (I^2^ = 48%) and 0.37 per cases on central nervous system (CNS) specimens (I^2^ = 0%). Detection of RSV within the CNS was relatively rare (17.86% of pooled case reports), being associated with male gender (adjusted odds ratio [aOR] 5.021, 95% confidence interval [95%CI] 1.104 to 22.831) and recovery with long-term sequelae (aOR 5.699, 95%CI 1.152; 28.183). Case fatality ratio was estimated to be 0.43 per 100 cases on observational studies and 10.71% in case reports, a difference likely due to publication bias. In summary, RSV represented a not frequent but notable cause of encephalitis/encephalopathy in adults and children. The paucity of available studies not only recommends a cautious appraisal of our results but stresses the clinical significance of future studies on incident cases of encephalitis and/or encephalopathy.

## 1. Introduction

Human respiratory syncytial virus (RSV; order mononegavirales, genus orthopneumovirus; family *Pneumoviridae*) is a pleomorphic, enveloped, single-stranded, negative-sense RNA virus (15 to 16 kb) of medium size (120–300 nm diameter) [1,2,3,4]. A highly contagious pathogen, RSV is associated with a high burden of lower respiratory tract infections (LRTI), particularly in infants aged 2 years or less [1,2,3] and in the elderly [5,6]. According to available estimates, before the COVID-19 pandemic, every year RSV was the single causative agent of around 33 million cases of acute respiratory infections (ARI) and lower respiratory tract disease (LRTD) [2,3,7], with a well-defined seasonal trend [2,7].

Notably, RSV causes high hospitalization rates in infants [8,9,10,11,12], even healthy children [13,14], without noticeable comorbidities [2,15,16], irrespective of their baseline clinical conditions [11,17,18,19]. Nonetheless, RSV also affects older individuals [15,20,21,22], where it causes high rates of respiratory illnesses and severe LRTI [23], particularly in adults aged 75 years or older [24,25], especially among those institutionalized [2,26,27,28] and/or characterized by pre-existing comorbidities (i.e., chronic obstructive pulmonary disease and congestive heart disorder) [22,29,30], with a substantial public health impact [31,32,33,34,35,36].

According to a recent summary of the literature, 1% to 7% of all children hospitalized with RSV infection develop some sort of neurologic complications, including encephalitis/encephalopathy [37], complex seizures, and status epilepticus [38]. The underlying mechanisms remain unclear [14,38]. On the one hand, there is considerable evidence that RSV can directly infect cells within the central nervous system (CNS) [39,40]. On the other hand, the considerable inflammatory response elicited by RSV infection within the respiratory tract could affect the CNS [41,42,43,44], either directly, through certain neuronal receptors (mostly GABA receptors), or indirectly, as a consequence of the high temperatures induced by the hypothalamic stimulation, with increased recycling of synaptic vesicles, their enlargement, and the eventual enhancement of synaptic transmission [39,41,42,43,45,46,47,48,49]. Moreover, severe cases of RSV are associated with substantial impairment of respiratory function and gas exchanges [9,11,50,51], which in turn could result in indirect damage to the CNS [52,53,54,55,56].

Because of the potential significance from a public health point of view, a systematic review synthetizing available reports on CNS complications of RSV infections can therefore be particularly useful to healthcare professionals potentially involved in the management of encephalitis/encephalopathies of unknown origin diagnosed during the RSV season or associated with respiratory infections. More precisely, we focused our analysis on cases of encephalitis and/or encephalopathy associated with RSV infections, undertaking a systematic review with meta-analysis aimed to ascertain the following: (a) whether RSV infections may be associated with an increased risk of encephalitis/encephalopathy, (b) whether RSV infections may result in CNS invasion, and (c) the potential outcome of RSV-associated encephalitis/encephalopathies.

## 2. Materials and Methods

### 2.1. Research Concept

This systematic review with meta-analysis was designed in accordance with the PRISMA (Prepared Items for Systematic Reviews and Meta-Analysis) statement [57,58]. As a preventive step, it was recorded in the PROSPERO (Prospective Register of Systematic Reviews) database with the ID number CRD42024534726 (Supplementary Table S1; https://www.crd.york.ac.uk/prospero/) (accessed on 10 April 2024) [59].

The research concept was defined through the “PICO” strategy (Patient/Population/Problem; Investigated results; Control/Comparator; Outcome) [60,61]. Briefly, the present study aimed to determine whether patients affected by RSV-related encephalitis/encephalopathy (P) with documented RSV detection within the CNS (I) compared with those without documented RSV detection within the CNS (C) are at higher risk for death or residual complications (O).

### 2.2. Research Strategy

Beginning on 10 April 2024, three scientific databases (i.e., PubMed, SCOPUS, and EMBASE) were searched for entries on RSV encephalitis in adults and children without any chronological restriction. A “snowball” approach was also implemented, with references of the retrieved studies and case reports analyzed for suitable entries not identified from the primary research option [62]. The detailed research strategy is reported in Annex Table A1.

### 2.3. Selection Criteria

Case reports, case series, cross-sectional studies, and case control studies with either a prospective or retrospective design written in English, Italian, German, French, or Spanish were considered suitable for inclusion in the meta-analysis. All articles were initially screened by their title for their relevance to the research subject [57,58]. All articles that were positively title-screened were then screened by the content of their abstracts. If considered consistent with the aims and the design of the present review, the full texts were independently assessed by two investigators (MB, SC) and abstracted.

### 2.4. Inclusion Criteria

In order to be included in the present systematic review, the studies reported on encephalitis and/or encephalopathy occurring in either children or adults. The following working definitions of encephalitis and encephalopathy were applied:

#### 2.4.1. Encephalitis

According to the International Encephalitis Consortium criteria [63], the main major criterion represented by an altered mental status lasting more than 24 h should be associated with at least two minor criteria, including fever for 72 h before the onset of symptoms, new onset seizures, new onset clinical features, white blood cells count > 5 mm^3^ in the cerebrospinal fluid (CSF), new neuroimaging findings, and abnormal electroencephalography (EEG) [63,64,65].

#### 2.4.2. Encephalopathy

Clinical case characterized by diffuse brain dysfunction or brain failure in either anatomical or functional features (or both) [66].

Reported encephalitis/encephalopathies were considered RSV-associated based upon the following: (a) the inception of CNS signs and/or symptoms occurred up to 8 days before and up to 21 days after the diagnosis of RSV infection [65,67,68]; (b) laboratory diagnosis of RSV infection was obtained in subjects having been diagnosed with encephalitis/encephalopathy within 15 days after the onset of the CNS signs and symptoms [63,65,69]. Only diagnoses of RSV infection on respiratory and/or CNS specimens by means of direct/indirect immunofluorescence (DIF/IIF), cell cultures, real-time quantitative polymerase chain reaction (RT-qPCR), and rapid antigen tests were included in the analyses.

Articles were excluded if they met the following criteria:

(a) The main text was not available in English, Italian, German, French, or Spanish;
(b) Information regarding clinical features and outcomes was insufficient;
(c) The study did not report the working definition for encephalitis and/or encephalopathy either extensively or by reference to official case definitions;
(d) Clinical criteria for the diagnosis of encephalitis and/or encephalopathy were not provided;
(e) Studies based on serology were eventually excluded as unable to ascertain whether the RSV infection was associated with reported neurological features or not.

### 2.5. Data Extraction

Data extracted by reviewers included (where available) the following:

(a) Details of the study: year, month or season, geographic region;
(b) Age and gender of the reported cases;
(c) History of prematurity (only cases < 14 years at the diagnosis, if available);
(d) Clinical characteristics at the onset of the symptoms; more precisely, the following signs and symptoms were taken into account: fever (body temperature > 37.8 °C); cough; wheezing; dyspnea and/or tachypnea; diagnosis of or symptoms associated with LRTI status (i.e., bronchitis, bronchiolitis, and pneumonia) and/or influenza-like illness (i.e., acute respiratory infection with fever and cough with onset within the previous 10 days) [14,70,71]; altered state of consciousness; disorientation; disorders of the eye movement; aphasia/slurred speech; ataxia and disorders of the gait; seizures; apnea;
(e) Features of imaging studies, including computed tomography (CT) and magnetic resonance imaging (MRI) studies, at the onset of clinical symptoms;
(f) Features of electroencephalographic studies; whether focal or general anomalies were reported; signs of slowed rhythm;
(g) Outcomes: intubation (with/without extra-corporeal membrane oxygenation, ECMO); cardiac arrest; survival (with and without any residual morbidity) vs. death.

Patients identified as cross-posted by different studies were accurately analyzed in order to fill the knowledge gaps and provide an extensive description of the clinical case as well as to eliminate duplicates.

### 2.6. Qualitative Assessment

Qualitative assessment of retrieved studies was performed according to Murad et al. [72] for case reports, and by means of a risk of bias (ROB) tool from the National Toxicology Program (NTP)’s Office of Health Assessment and Translation (OHAT) (now the Health Assessment and Translation, HAT, group) [73,74] for cross-sectional studies and case-control studies.

#### 2.6.1. Case Reports/Case Series

In their study, Murad et al. [72] proposed to rate case reports and case series in four main domains (selection, ascertainment, causality of case, and reporting quality), through a total of eight dichotomous (“high risk” vs. “low risk”) items, including the following:

D1. “Does the patient(s) represent(s) the whole experience of the investigator (centre) or is the selection method unclear to the extent that other patients with a similar presentation may not have been reported?”
D2. “Was the exposure adequately ascertained?”
D3. “Was the outcome adequately ascertained?”
D4. “Were other alternative causes that may explain the observation ruled out?”
D7. “Was follow-up long enough for outcomes to occur?”
D8. “Is the case(s) described with sufficient details to allow other investigators to replicate the research or to allow practitioners to make inferences related to their practice?”

On the contrary, two items specifically designed for reports on adverse drug events (D5, “Was there a challenge/rechallenge phenomenon?” and D6, “Was there a dose–response effect?”), were eventually included.

In accordance with the original recommendations from Murad et al. [72], even studies with “high” or “unclear risk” ratings in one or more of the assessed domains were included in the eventual body of evidence.

#### 2.6.2. Cross-Sectional Studies/Case-Control Studies

The OHAT ROB tool evaluates the internal validity of a given study by means of the following potential sources of bias [73,74]:

(1) Selection bias (“Did selection of study participants result in appropriate comparison groups?”);
(2) Confounding bias (“Did the study design or analysis account for important and modifying variables?”);
(3) Exclusion bias (“Were outcome data complete without attrition or exclusion from analysis?”);
(4) Detection bias (“Can we be confident in the exposure characterization?” and “Can we be confident in the outcome assessment?”);
(5) Selective reporting bias (“Were all measured outcomes reported?”);
(6) Other sources of bias (“Were there no other potential threats to internal validity (e.g., statistical methods were appropriate, and researchers adhered to the study protocol)?”).

All potential sources of bias are then rated through a four-point scale ranging from “definitely low”, “probably low”, “probably high”, to “definitely high”. However, by its design, OHAT ROB does not provide a cumulative rating for each study as it requires that even studies affected by a certain degree of ROB be included in the pooled analyses in order to provide an extensive appraisal of the retrieved literature [74].

#### 2.6.3. Handling of Individual Scores

The full-text versions of all eligible articles were independently read and then rated according to the aforementioned scoring systems by two investigators (MB and SC). Disagreements were initially managed by searching the consensus between the two reviewers. Where it was not reached, the chief investigator (MR) was involved as a third person.

### 2.7. Data Analysis

#### 2.7.1. Descriptive Analysis

Included studies were summarized by descriptive analysis, separately for observational studies and case control/case series. Crude prevalence figures as per 100 people were therefore calculated. The distribution of categorical variables was initially analyzed by the documented identification of RSV within CNS and/or CSF through a chi-squared test with continuity correction. Continuous variables were initially checked for normality distribution by means of a K2 test and then either assessed by means of the Student’s *t* test for unpaired data for *p* value > 0.100 (i.e., normally distributed) or Mann–Whitney tests for *p* value < 0.100 (i.e., not normally distributed). All variables that in univariable analysis were associated with the identification of RSV within the CNS with *p* < 0.250 were included in a multivariable regression analysis model. Their association with the outcome variable was eventually reported as for adjusted odds ratio (aOR) with their 95% confidence intervals (95%CI).

#### 2.7.2. Diagnostic Accuracy

The accuracy of CT, MRI, and EEG in the identification of RSV infections within CNS/CSF was measured by calculating the corresponding sensitivity (Se), specificity (Sp), and positive and negative predicted values (PPV and NPV) compared to laboratory studies.

#### 2.7.3. Meta-Analysis

Meta-analysis of observational studies was performed through a random-effect model (REM) on the prevalence data of RSV infections and reported clinical features. Eventual estimates were reported with their 95% confidence intervals (95%CI). REM was preferred over a fixed-effect model as being considered more effective in dealing with the genuine differences underlying the results of the studies (heterogeneity) [75,76]. The presence of variation in true effect sizes (i.e., heterogeneity) was defined as the percentage of total variation across studies likely due to clinical differences, methodological issues, absolute rather than relative measures of risk, and publication bias rather than chance [77] and was quantified by I^2^ statistic through the following categories: 0 to 25%, low heterogeneity; 26% to 50%, moderate heterogeneity; ≥50%, substantial heterogeneity. In order to cope with the potentially small number of retrieved studies and collected cases, which would likely cause underestimation of actual heterogeneity when only reported as point estimates of I^2^, 95%CI were provided [77].

Sensitivity analysis (i.e., the study of how the uncertainty in the output of a mathematical model or system can be apportioned to different sources of uncertainty in its inputs) was performed to evaluate the effect of each study on the pooled estimates by excluding one study at a time.

Publication bias was initially assessed through calculation of contour-enhanced funnel plots. Their asymmetry was initially visually assessed and then ascertained by calculation of the Egger’s test for all outcomes with three or more included studies [57,78]. Small study bias was assessed by generating corresponding radial plots. A *p* value < 0.05 was considered statistically significant for both publication and small study bias.

Screening of retrieved articles was performed on Mendeley Reference Manager (version 2.112.2; Mendeley Ltd.; New York, NY, USA). Calculations were performed in R (version 4.3.0) [79] and RStudio (version 2023.03.0; RStudio, PBC; Boston, MA, USA) software by means of the packages “meta” (version 6.5-0), “fmsb” (version 0.7.6), “epiR” (version 2.0.73), and “robvis” (version 0.3.0). Plots were calculated by means of R packages “ggplot2” (version 3.5.0), “ggpubr” (version 0.6.0), and “PRISMA2020” (version 1.1.1) and GraphPad Prism, version 10.2.2 (GraphPad Software LLC, Boston, MA, USA).

## 3. Results

### 3.1. Descriptive Analysis

**Figure 1:**
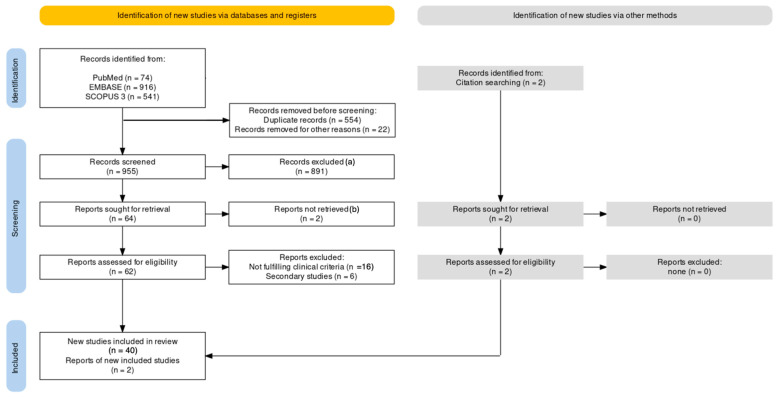
Flowchart of 1531 studies included in the present systematic review. Note: other reasons = studies reported in languages other than those understood by authors of the present study (English, Italian, German, French, or Spanish); a: studies not fulfilling inclusion criteria; b: studies not retrieved through medical databases and/or interlibrary loan.

As shown in Figure 1 and Annex Table A1, a total of 1531 studies were initially identified, most of them from EMBASE (n = 916, 59.83%), followed by Scopus (n = 541, 35.34%), with 74 from PubMed (4.83%). After the removal of reports duplicated across the searched databases (n = 554, 36.19%) or reported in languages other than those understood by authors of the present study (n = 22, 1.44%), a total of 955 articles were title- and abstract-screened.

Of them, 891 were considered not consistent with the research strategy and inclusion criteria and were then removed from the analyses. In contrast, 64 entries (4.18% of the original sample) were sought for retrieval: 62 of them were ultimately made available through medical databases and/or interlibrary loan and were assessed for eligibility, with the resulting exclusion of 15 papers that did not fulfill inclusion criteria or that were secondary studies. A total of 40 entries were therefore included in the analyses (2.61% of the original sample), with two further entries identified by means of the snowball approach [69,80].

### 3.2. Observational Studies

Overall, 15 entries included observational studies [52,53,55,69,80,81,82,83,84,85,86,87,88,89,90], and their main characteristics are summarized in Table 1.

#### 3.2.1. Prevalence Estimates

More precisely, 7 out of aforementioned 15 studies reported the occurrence of encephalitis or encephalopathy cases on diagnoses of RSV infections, and their characteristics are detailed in Table 2 [52,53,55,81,82,83,84].

Briefly, four studies were performed in the USA [52,53,55,81], with three further studies from South Korea [84], Turkey [92], and the United Kingdom [82]. Mirroring the broad timeframe of the parent studies (ranging from 1994 to 2016), diagnosis of RSV infection was performed by means of either IIF + viral cultures [52,81,82] or RT-qPCR [53,55,83,84]. A total of 7719 cases of RSV infection were collected (individual size of studies ranging from 130 to 3856), including 198 cases of either encephalopathy/encephalitis, with prevalence estimates ranging from 0.21% [83] to 39.2% [53] (crude estimate of 2.57% for the whole of retrieved cases).

**Figure 2:**
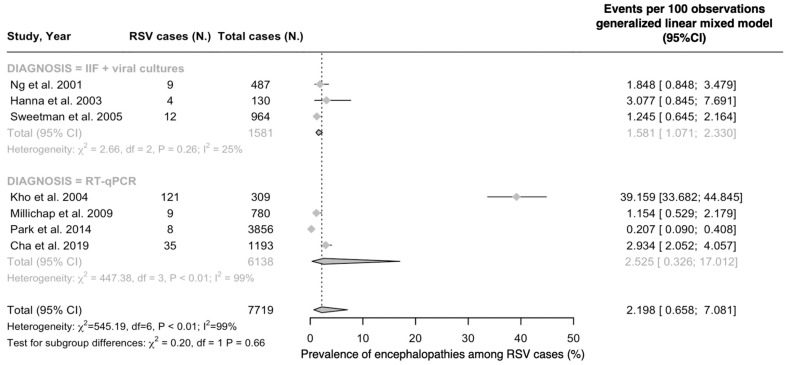
Prevalence of encephalopathies among cases of respiratory syncytial virus (RSV) infections (% value). Note: 95%CI = 95% confidence intervals; IIF = indirect immunofluorescence; RT-qPCR = real-time quantitative polymerase chain reaction [52,53,55,81,82,83,84].

In the REM model (Figure 2), a pooled estimate of 2.20% (95%CI 0.66 to 7.08) was calculated, with no significant differences for studies based on IIF and viral cultures (1.58%, 95%CI 1.07 to 2.33) and RT-qPCR (2.53%, 95%CI 0.33 to 17.01; chi squared = 0.20, *p* = 0.66).

Pooled estimates were affected by substantial heterogeneity (I^2^ = 98.9%, 98.5 to 99.2, tau^2^ = 2.609; *p* < 0.001), particularly for studies based on RT-qPCR (I^2^ =99.3%) compared to those based on IIF + viral cultures (I^2^ = 24.9%).

Focusing on articles reporting the proportion of RSV cases over the whole of diagnosis of encephalitis/encephalopathy, a total of eight studies were identified [69,80,85,86,87,88,89,90] (Table 3), ranging from 2000 to 2020, mostly based on RT-qPCR, with two further studies based on IIF [88,89]. Overall, 1631 cases of encephalitis/encephalopathy were retrieved [69,80,85,86,87,90], including a total of 6 cases where RSV was identified from CSF samples [69,80,85,86,87,88,89,90], for a crude prevalence of 0.49% (Table 2). In 14 cases from three studies including a total of 419 patients [87,88,89], RSV was identified from respiratory specimens (crude prevalence of 3.34%).

**Figure 3:**
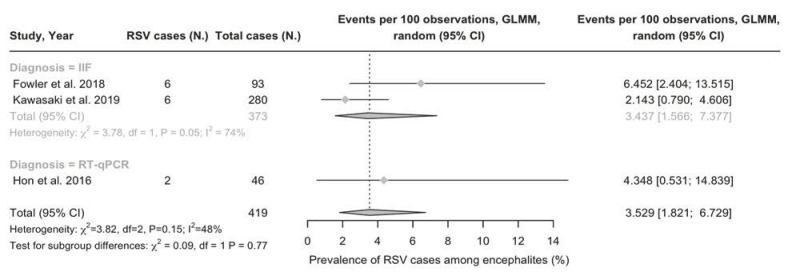
Proportion of cases of respiratory syncytial virus (RSV) infection among cases of viral encephalopathies (% value), virus detected from respiratory specimens. Note: 95%CI = 95% confidence intervals; IIF = indirect immunofluorescence; RT-qPCR = real-time quantitative polymerase chain reaction [87,88,89].

**Figure 4:**
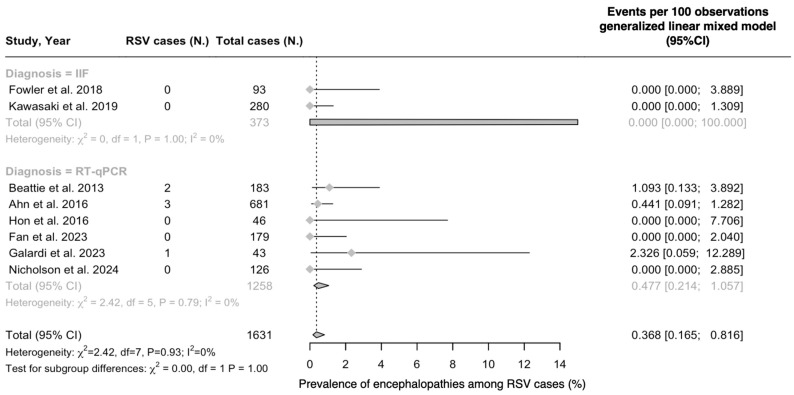
Proportion of cases of respiratory syncytial virus (RSV) infection among cases of viral encephalopathies (% value), virus detected within the cerebrospinal fluid. Note: 95%CI = 95% confidence intervals; IIF = indirect immunofluorescence; RT-qPCR = real-time quantitative polymerase chain reaction [69,80,85,86,87,88,89,90].

Corresponding pooled prevalence estimates from REM are reported in Figure 3 and Figure 4. More precisely, a pooled prevalence of 3.53% (95%CI 1.82 to 6.73) was calculated on respiratory specimens (Figure 3). The point estimate hinted at a moderate heterogeneity (47.7%; tau^2^ = 0.100; *p* = 0.148) even though 95%CI estimates of I^2^ rather suggested that results were affected by substantial heterogeneity (95%CI 0.0 to 84.7).

On the other hand, a pooled prevalence of 0.37% (95%CI 0.17 to 0.82) was calculated from analysis of CSF or brain specimens, and even though results were seemingly not affected by residual heterogeneity (I^2^ = 0.0%; tau^2^ = 0, *p* = 0.933), residual heterogeneity was suggested by 95%CI (0.0 to 67.6). No cases were reported from studies based on IIF, while all six positive cases were identified by means of RT-qPCR.

#### 3.2.2. Clinical Characteristics

Clinical characteristics of RSV cases associated with encephalitis/encephalopathy are provided in Table 4. Because of the heterogeneous reporting strategy, only the occurrence of seizures was calculated over 210 total cases. However, available data suggest that most cases were of male gender (60.33%, 95%CI 48.47 to 71.10), aged less than one year at the onset of the reported diagnosis of encephalitis/encephalopathy (57.02%, 95%CI 36.48 to 75.40), and affected by prematurity (52.94%, 95%CI 36.46 to 68.81). Nearly all cases reporting clinical features showed respiratory signs and symptoms belonging to the definition of LRTI (99.55%, 95%CI 58.04 to 100). Around half of the cases developed apnea (46.6%; 95%CI 0.0 to 80.4) and/or required intubation because of their respiratory disorders (55.1%; 95%CI 0.0; 83.4). Seizures were reported in most cases (83.96%, 95%CI 33.07; 98.23), while only 1.00% (95%CI 0.02 to 33.47%) developed cardiac arrest. Even though only the point estimate for the prevalence of seizures breached the cut-off value for substantial heterogeneity (71.2%; 95%CI 43.2; 85.4), the analysis of 95%CI suggested that the residual heterogeneity should have been considered for the remaining analyses.

.

**Figure 5:**
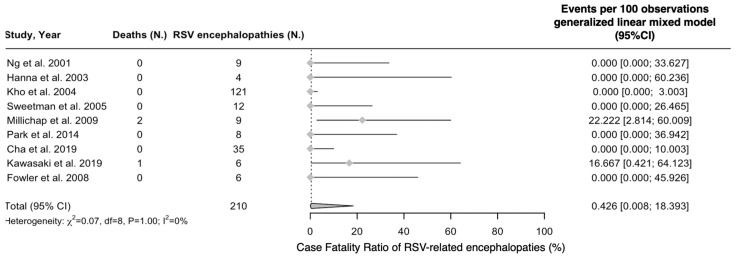
Case fatality ratio (CFR) of respiratory syncytial virus (RSV) infections among cases of viral encephalopathies (% value). Note: 95%CI = 95% confidence intervals; IIF = indirect immunofluorescence; RT-qPCR = real-time quantitative polymerase chain reaction [52,53,55,81,82,83,84,88,89].

Regarding the outcome of the RSV-related encephalitis/encephalopathies, a total of three deaths were eventually reported from the whole of 210 cases, with a crude case fatality ratio (CFR) of 1.43% and pooled REM estimate of 0.43%, 95%CI 0.01 to 18.39 (Figure 5). Overall, the heterogeneity of pooled estimates was considered low (I^2^ = 0.0%), but 95%CI hinted at a noticeable residual heterogeneity (0.0 to 64.8).

#### 3.2.3. Diagnostic Features

As shown in Table 5, EEG studies showed the highest proportion of anomalies (60.21%, 95%CI 38.80 to 78.31), followed by MRI (20.62%, 12.33 to 32.41) and CSF analysis (12.88%, 95%CI 1.32 to 62.04), while CT studies of the brain and brainstem were less likely to be associated with any specific finding (9.23%, 95%CI 0.73 to 58.48). Even though point estimates suggested that pooled estimates were only limitedly affected by heterogeneity, analysis of 95%CI was less favorable, as only for MRI studies did the higher limit not exceed the cut-off value for substantial heterogeneity (I^2^ = 0.0%, 95%CI 0.0 to 67.6).

### 3.3. Summary of Case Reports and Case Series

#### 3.3.1. Clinical Features

A total of 27 case reports were ultimately identified [91,93,94,95,96,97,98,99,100,101,102,103,104,105,106,107,108,109,110,111,112,113,114,115,116,117,118]. As five observational studies included clinical data that allowed the appropriate characterization of individual cases [52,55,81,83,119], a total of 84 cases of RSV-related encephalopathy/encephalitis were ultimately included in the analyses. Their individual features are summarized in Table 6.

Overall, the majority of cases were of male gender (55.96%), with a mean age of 4.4 years ± 11.7, but nearly half of subjects were aged less than one year at the time of the onset of symptoms (46.43%). Prematurity was reported in only 9.5% of cases. Regarding their clinical features, the most frequently reported signs/symptoms included seizures (65.48%), followed by body temperature > 37.8 °C (48.81%), altered state of consciousness including dizziness and coma (42.86%) with 14.29% affected by disorientation, cough (38.10%), dyspnea/tachypnea (26.19%), and anomalies of the eye movement (15.48%). Only 13.10% of cases were affected by ataxia and 7.14% by aphasia or impairment of the language, and 11.90% of cases developed apnea. In summary, 27.38% of cases exhibited signs and symptoms associated with the diagnosis of ILI, while 14.29% were affected by LRTI. Regarding the outcome of the reported cases, 13.10% developed cardiac arrest, 27.38% required intubation, and 2.38% required treatment with ECMO. In around 1/3 of cases (32.14%), either the case was lost to follow-up or the eventual status of the patient was not reported. Regarding the remaining cases, 34.52% had a full recovery, 22.62% recovered from the RSV-related encephalitis/encephalopathy with any residual impairment, and 10.71% died because of the consequences of RSV-related syndrome.

Respiratory syncytial virus was identified within CSF or brain specimens in 15 cases (17.9%), and this finding was associated with an altered status of consciousness (73.3% vs. 36.2% among those without RSV within CSF or brain specimens; *p* = 0.019) and reporting signs/symptoms associated with an ILI (53.3% vs. 21.7%; *p* = 0.030). However, when the identification of RSV within CNS/CSF was associated with a *p-value* < 0.250 with age < 1 year (*p* = 0.159), male gender, (*p* = 0.227), fever (*p* = 0.214), cardiac arrest (*p* = 0.195), and the eventual status of the patient (*p* = 0.081), multivariable analysis was modelled accordingly.

As shown in Table 7, documenting RSV within the CNS was eventually associated with cases of male gender (aOR 5.021, 95%CI 1.104 to 22.831) and having a partial rather than a full recovery (aOR 5.699, 95%CI 1.152 to 28.183).

#### 3.3.2. Diagnostic Features

The most frequently reported diagnostic study was represented by MRI (66.67%), followed by CT (54.76%) and EEG (46.43%). Pathological findings were identified in 79.49% of performed EEG, followed by MRI (73.21%), and CT (47.83%) (Table 8). The highest sensitivity was reported by EEG studies (1.000, 95%CI 0.610 to 1.000) followed by MRI (0.900, 95%CI 0.596 to 0.995), with the lowest performances from CT studies (0.455, 95%CI 0.212 to 0.720). In turn, CT studies had better specificities (0.514, 95%CI 0.356 to 0.670) followed by MRI (0.304, 95%CI 0.191 to 0.670), and the lowest performance was from EEG (0.242, 95%CI 0.128 to 0.430). Therefore, PPV of CT (0.227, 95%CI 0.101 to 0.434), MRI (0.220, 95%CI 0.120 to 0.367), and EEG (0.194, 95%CI 0.092 to 0.363) were largely unsatisfying. Conversely, high estimates of PNV were associated with all diagnostic options and particularly with EEG (1.000, 95%CI 0.676 to 1.000) and MRI (0.933, 95%CI 0.702 to 0.997), followed by CT studies (0.750, 95%CI 0.551, 0.880).

Specific features of MRI studies are detailed in Table 9. In fact, in 30.36% of cases, the affected site was not reported. Regarding the remaining cases, the most common finding was signs of diffuse damage (41.07%), followed by diffuse damages within the neocortex (23.21%), with 12.5% of cases affected within the frontal cortex, 10.71% in the temporal cortex, and 5.36% in the occipital cortex. The cerebellum was associated with pathological findings in 16.07% of cases, while the mesencephalon was reportedly affected in 8.93% of all studies. Eventually, pathological findings were also reported from the basal ganglia (14.29%) and corpus callosum (7.14%). Documenting RSV within the brain and/or CSF was only associated with findings from the frontal cortex (40.00% vs. 6.5%, *p* = 0.018).

Regarding EEG studies (Table 10), 20.51% of cases were negative, while the majority of the remaining studies were affected by interictal epileptiform discharges (43.59%), with the remaining cases affected by non-epileptiform abnormalities (35.90%), with no substantial differences between cases with and without documented RSV infection within the CNS (*p* = 0.311).

### 3.4. Risk of Bias

**Figure 6:**
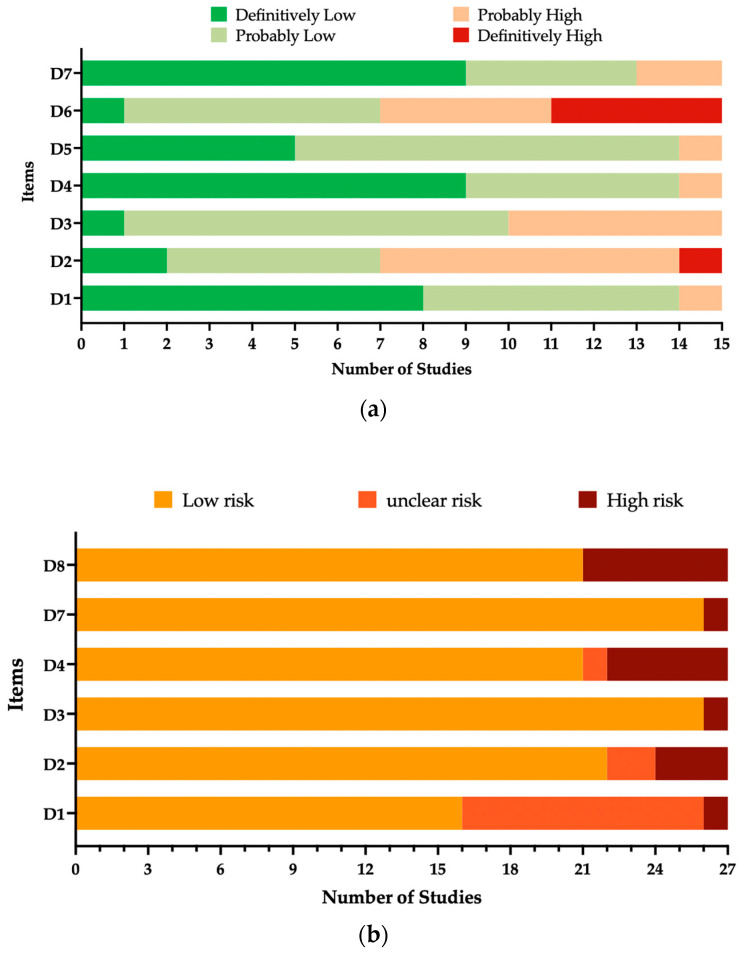
Summary of risk of bias analysis, from (**a**) observational studies (performed according to the risk of bias tool from the National Toxicology Program’s Office of Health Assessment and Translation handbook [74,120]); (**b**) case reports of respiratory syncytial virus encephalopathy (performed according to Murad et al. [72], modified).

The overall quality of the included observational studies is summarized in Figure 6a, while a summary of case reports is provided in Figure 6b. Despite heterogeneities in design and reporting strategy, all studies were reasonably characterized by high quality and low risk of bias. Individual data are included in Appendix A, Table A2 and Table A3.

### 3.5. Sensitivity Analysis

**Figure A1:**
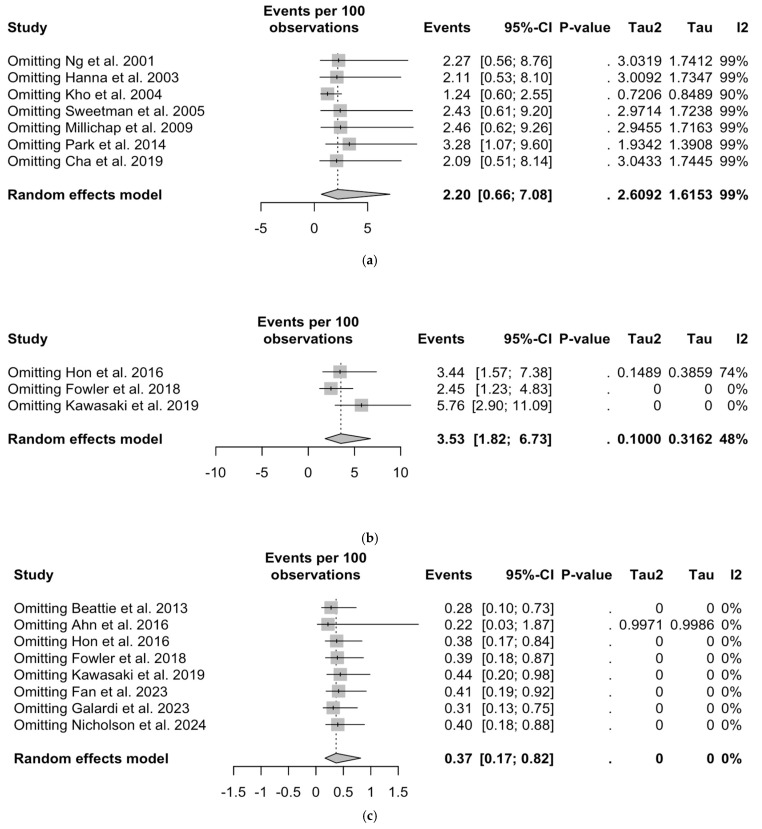
Sensitivity analysis (**a**); occurrence of respiratory syncytial virus (RSV) infections among series of encephalitis, diagnosis on respiratory specimens (**b**); occurrence of encephalitis among cases of RSV infections (**c**). Sensitivity analysis was performed by omitting from the pooled estimates one study at time [52,53,55,69,80,81,82,83,84,85,86,87,88,89,90].

**Figure A2:**
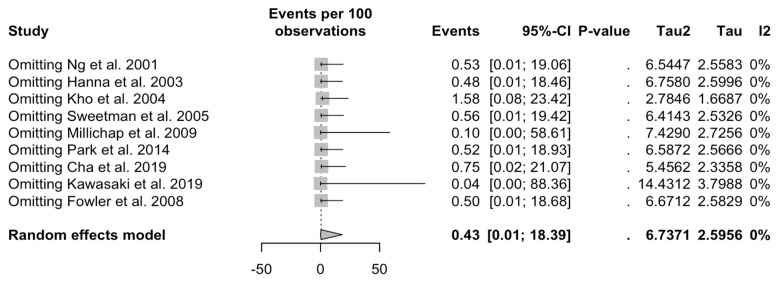
Sensitivity analysis for case fatality rate of respiratory syncytial virus associated encephalopathy. Sensitivity analysis was performed by omitting from the pooled estimates one study at time [52,53,55,81,82,83,84,88,89].

Sensitivity analysis required the removal of single studies at a time, and the resulting pooled estimates are reported in Appendix A, Figure A1 and Figure A2. When dealing with the proportion of RSV cases over the total of reported diagnoses of encephalitis or encephalopathy (Appendix A, Figure A1a) from respiratory specimens, the removal of the studies of Kho et al. [53] led to a reduced prevalence estimate (1.24%, 95%CI 0.61 to 9.20), while by omitting the study of Park et al. [83], prevalence estimates increased to 3.28% (95%CI 1.07 to 9.60). Focusing on studies for RSV cases complicated by encephalitis/encephalopathies with diagnosis on CSF (Appendix A, Figure A1b), the removal of Fowler et al. [88] led to a reduction in pooled estimates (2.45%, 95%CI 1.23 to 4.83), while omitting Kawasaki et al. [89] increased the estimate to 5.76% (95%CI 2.90 to 11.09). Regarding the studies on encephalitis/encephalopathies with a diagnosis on respiratory specimens (Appendix A, Figure A1c), only the removal of the study of Ahn et al. [86] resulted in a reduced estimate (0.22%, 95%CI 0.03 to 1.87), while omitting Kawasaki et al. [89] increased the estimate to 0.44% (0.20 to 0.98).

Focusing on the pooled case fatality rate (CFR, Appendix A Figure A2), while no substantial differences were identified in terms of heterogeneity, the removal of Kho et al. [53] increased the estimate to 1.58% (95%CI 0.08 to 23.42), while the omission of Millichap et al. [55] reduced the estimate to 0.10% (95%CI 0.00 to 58.61) and that of Kawasaki et al. [89] to 0.04% (95%CI 0.00 to 88.36).

### 3.6. Publication Bias

**Figure 7:**
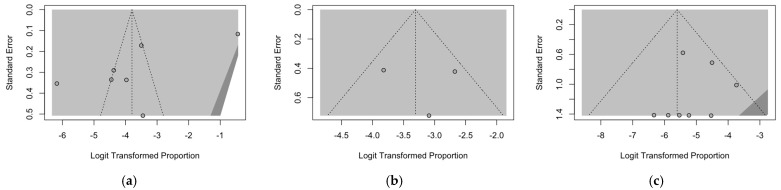
Funnel plots on observational studies, reporting on the occurrence of RSV (respiratory syncytial virus) infections among series of encephalitis, diagnosis on cerebrospinal fluid (**a**); occurrence of RSV infections among series of encephalitis, diagnosis on respiratory specimens (**b**); occurrence of encephalitis among cases of RSV infections (**c**).

As a preliminary step, publication bias was ascertained by calculation and visual inspection of funnel plots. In funnel plots, the sample size is plotted against the effect size: as the size of the sample increases, individual estimates of the effect likely converge around the true underlying estimate [63,66,73]. Funnel plots for prevalence estimates are reported in Figure 7.

**Figure 8:**
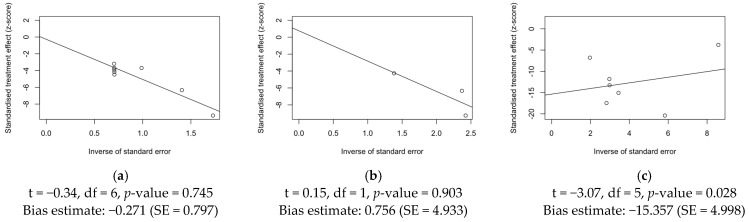
Radial plots on observational studies, reporting on the occurrence of respiratory syncytial virus (RSV) infections among series of encephalitis, diagnosis on cerebrospinal fluid (**a**); occurrence of RSV infections among series of encephalitis, diagnosis on respiratory specimens (**b**); occurrence of encephalitis among cases of RSV infections (**c**). Results of corresponding linear regression test for funnel plot asymmetry (i.e., Egger’s test) are reported within each subfigure. Note: SE = standard error).

Taking into account the reduced sample size, all funnel plots were substantially asymmetrical, suggesting the presence of publication bias. However, Egger’s test (Figure 8) substantially ruled out potential publication bias for prevalence estimates of RSV infection over cases of encephalitis/encephalopathies (Figure 8a) and cases of RSV infection affected by encephalitis/encephalopathies and based on respiratory specimens (Figure 8b), suggesting that other potential sources of asymmetry (e.g., heterogeneity due to different choices in the outcome measure, differences in the underlying risk, etc.) should be ascertained. On the other hand, for studies based on cases of RSV infection affected by encephalitis/encephalopathies and CSF specimens (Figure 8c), publication bias was confirmed by Egger’s test (*p* = 0.028).

**Figure 9:**
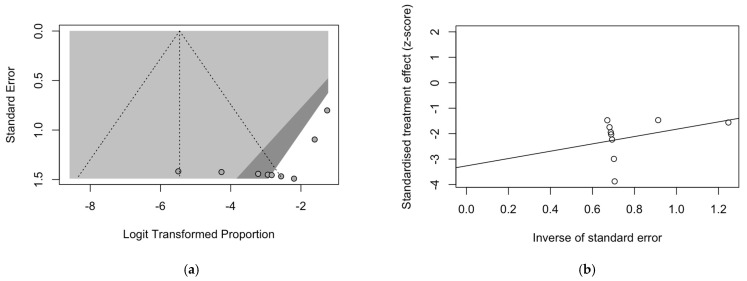
Funnel plot for the meta-analysis of case fatality ratio among cases of respiratory syncytial virus-associated encephalitis (**a**). Corresponding radial plot (**b**). Linear regression test for funnel plot asymmetry (t = −2.75, df = 7, *p*-value = 0.029; bias estimate: −3.267; standard error = 1.190).

An analysis of publication bias on studies reporting CFR is shown in Figure 9. Again, visual inspection of the funnel plot suggests the presence of substantial publication bias, which was confirmed by Egger’s test (*p* = 0.029).

## 4. Discussion

### 4.1. Summary of Main Findings

In this systematic review with meta-analysis, we collected available data from observational studies and case reports/case series on RSV infection and encephalitis/encephalopathies. Observational studies reporting on encephalitis/encephalopathies identified a pooled prevalence of RSV infections equal to 2.20% (95%CI 0.66 to 7.08). On the other hand, observational studies reporting on series of RSV infections hinted at a pooled prevalence of 3.53% (95%CI 1.82 to 6.73) for studies based on respiratory specimens only and 0.37% (0.17 to 0.82) for studies based on CSF analysis. In other words, not only were RSV infections characterized as a relatively rare finding when dealing with encephalitis/encephalopathies but also, in turn, encephalitis and encephalopathies were identified as relatively rare complications of RSV infections. Moreover, estimates of CFR from observational studies hinted at a low risk of death following RSV infection with neurologic complications, as only three deaths were reported over a total of 210 cases (CFR 0.37%; 95%CI 0.17 to 0.82).

Pooled data from case reports and case series led to a quite different estimate, as nine deaths were reports among 84 cases of RSV-related encephalitis, with a far larger risk of death (10.4%). Moreover, collected data stressed how long-lasting the consequences of RSV-related encephalitis/encephalopathies may be, as around 1/3 of all patients developed long-term sequelae, and this finding was significantly associated with documenting RSV infection within the CSF (aOR 5.699; 95%CI 1.152; 28.183)

### 4.2. Generalizability of the Results

Collectively, our data suggest that RSV can actually cause a noticeable proportion of all encephalitis/encephalopathies reported in the general population and particularly in subjects in the pediatric age group aged less than one year. According to the available estimates on RSV-related hospitalizations [6,10,121,122,123], in Germany alone (average of around 20,530 cases by year) [123], a total of 451.36 cases of RSV-related encephalitis/encephalopathies would occur (95%CI 135.12 to 1545.10), with around 19 deaths (95%CI 6 to 62). From a broader point of view, i.e., by taking into account the 158,229 RSV-associated hospitalizations recently estimated by Osei-Yeboah et al. [6] for adults in the European Union and the 245,244 RSV-associated hospitalizations similarly estimated by Del Riccio et al. for children aged less than five years [5], every year around 8868 hospitalizations due to RSV-related encephalitis/encephalopathies would occur (95%CI 2655 to 28,569), with around 378 deaths (95%CI 113 to 1217).

Even though several other respiratory pathogens have a documented neuroinvasive potential, with viral encephalitis representing a potential complication of respiratory tract infections [86,88,91,124,125], most of the reported cases are reasonably associated with indirect effects of viral infection rather than being due to RSV neurotropism, as previously suggested by studies on febrile seizures associated with RSV infections [38,39,84,126,127,128]. In fact, RSV was only detected from the CNS in 15 out of 84 collected case reports and in 6 out of 1631 cases pooled from observational studies (i.e., 17.86% vs. 0.37%). These differences could be explained through the nature and aims of case reports and case series. Case reports and case series are descriptive studies that illustrate novel, unusual, or atypical features identified in patients not systematically encountered during medical practice [129]. As a consequence, these studies usually oversample cases characterized by uncommon clinical features, including more severe outcomes. Therefore, substantial disagreement remains on the value of these studies in the medical literature, particularly for the collection of evidence [72,129]. In other words, the higher rate of RSV infection of the CNS from case reports compared to observational studies is likely due to either a selection bias or a confirmation bias.

The limited neuroinvasive potential usually exhibited by RSV could be otherwise explained through its biology. While pathogens such as influenza virus, coronaviruses, human metapneumovirus, adenovirus, and enterovirus exhibit an even transitory phase of viremia [65,69,130], after its entry through the respiratory tract, RSV spreads to the remainder of the respiratory epithelium through cell-to-cell transfer or the aspiration of nasopharyngeal secretions, with the resultant involvement of the airways as a whole [131,132,133]. Not coincidentally, while the proportion of cases of LRTI was similar in patients with and without documented RSV infection within the CSF (20.00% vs. 13.04%, *p* = 0.771), this finding was more frequently reported in cases with clinical features of ILI (53.33% vs. 21.74%, *p* = 0.030), suggesting a different course of the main infection with a more limited invasion of the lower respiratory tract.

### 4.3. Implications for Medical Practice

Despite the potential oversampling of severe cases, the potentially high rate of cases with long-term sequelae and their subsequent direct and indirect costs [35,36,134,135] collectively stress the need for appropriate preventive interventions. In fact, limited therapeutic options for RSV are currently available (including non-specific antiviral drugs such as ribavirin), and the management of incident infections mostly relies on symptomatic therapy. On the contrary, several preventative options have been made progressively available, starting with the monoclonal antibody (mAb) palivizumab (SYNAGIS^®^, Abbott Laboratories Ltd., Queenborough, Kent, UK) [136,137,138,139,140]. Despite its proven efficacy, due to the high costs associated with a full preventive course [141], palivizumab was only delivered to infants considered at high risk for RSV-related complications [14,142,143,144]. While the majority of cases included in this study reasonably occurred in patients who did not undergo such prophylactic treatment because of their underlying clinical and demographic features [14,145,146], the prevention of RSV-associated encephalitis/encephalopathy could otherwise benefit from nirsevimab, a longer half-life mAb that has been shown as quite effective in preventing severe cases of RSV both in preterm and in term infants [147,148,149,150,151,152,153,154,155,156,157], and from two preventive vaccines eventually licensed for human use (Abrysvo from Pfizer Inc., and Arexvy from GlaxoSmithKline LLC) [158,159]. On the one hand, early estimates on the real-world effectiveness of nirsevimab suggest that this mAb could effectively reduce the occurrence of severe complications in children aged <2 years [160,161,162,163], reducing, in turn, the likelihood of encephalopathies due to respiratory distress. On the other hand, both available vaccines have been documented as effective in preventing complications in adults older than ≥60 years [164,165], potentially providing a similar protective effect, and Abrysvo has been also licensed to be delivered in pregnant women to protect their offspring through maternal vaccination.

### 4.4. Limitations and Implications for Future Studies

Despite their potential significance from a clinical and public health perspective, our results are affected by substantial limits. First and foremost, despite the relatively high quality of the collected studies, the total number of reported cases of encephalitis/encephalopathy was low compared to the global estimates on RSV and viral encephalitis [65,86,88,91,130] with resulting doubtful representativity when dealing with both prevalence estimates and analysis of clinical and diagnostic features. More precisely, this systematic review collected data from 84 individual cases of encephalitis/encephalopathies associated with RSV infection and identified 198 cases complicated by neurological findings of encephalitis/encephalopathy from 7719 cases of RSV infection and 1631 cases of encephalitis/encephalopathy that in turn encompassed no more than 20 cases of RSV infection. On the other hand, according to the available estimates [1,2,166], every year RSV causes around 33 million cases of LRTI. Even the diagnosis of encephalitis due to viral pathogens represents a quite common finding: according to the recent review from Wang et al. [64], in 2019 alone the diagnosis of encephalitis was associated with 1,444,720 incident cases and 89,900 deaths at the global level.

Second, the diagnosis of encephalitis is mostly based on clinical criteria [63,64,65], and the working definition of encephalopathy is even more vague, representing an “umbrella term” that includes both anatomical and functional features (i.e., diffuse brain dysfunction or brain failure) [66]. Even though we tentatively limited the potential inclusion of cases not properly fitting with the aims of this systematic review [167,168,169], we cannot rule out the potential omission or even the oversampling of cases from the earlier studies [52,53,55,81,82].

Third, even though encephalopathy has been acknowledged to be among the potential consequences of severe RSV infections [38,54,55,170], mostly due to the respiratory and hypoxic complications of severe LRTI, the neuroinvasive potential of this pathogen has been mostly overlooked. Therefore, cases of encephalitis did not systematically inquired about the potential of RSV infection, particularly when the clinical features are not consistent with the suggestive status of LRTI [65,88]. Even when RSV was systematically investigated among the potential causes of viral encephalitis, results have been quite conflicting. For example, in the study of Fowler et al. [88], RSV accounted for a total of 7 out 93 cases of pediatric encephalitis, that is, 7.53% of the total sample, a proportion comparable to pathogens more likely associated with neuroinvasion, such as tick-borne encephalitis virus (8.60%), enterovirus (7.53%), varicella zoster virus (5.38%), and herpes simplex virus (2.15%). On the contrary, in the recent report from Nicholson et al. [90], all 107 plasma samples and 62 CSF samples re-collected from a total of 126 cases of pediatric encephalitis were ultimately negative for RSV. In this regard, it should be stressed that a large number of RSV cases are usually not properly diagnosed, even severe ones otherwise requiring hospitalization [5,6]. RSV is mostly considered a pediatric-age pathogen [171,172], and adults are therefore not consistently sampled for this pathogen, even when belonging to high-risk groups [173,174], with resulting underestimation of its actual occurrence. A potential strategy for reconciling with the misdiagnosis of RSV infections may be to include this pathogen in all clinical and laboratory workflows performed on cases of suspected viral encephalitis and encephalopathies of uncertain cause, particularly during the “RSV season”.

Fourth, even when properly sampled, estimates for RSV occurrence may be affected by substantial inaccuracy due to its pathology and the natural history of RSV infection, in accordance with the stage of the assessed infection [175,176]. Usually, RSV is sampled on respiratory specimens and particularly nasal swabs through RT-qPCR, usually considered the reference laboratory study for diagnosis of viral respiratory infections [177,178,179]. However, the role of RT-qPCR as the gold standard study for RSV has been extensively debated, and samples from the upper respiratory tract may show a falsely negative result in cases characterized by LRTI [180,181,182], as the viral infection has eventually “migrated” to the lower regions of the respiratory tract, being otherwise cleared from the upper tract [176]. Therefore, we cannot rule out that several cases of RSV-associated encephalitis may have been improperly diagnosed, being lost to the eventual estimates of individual studies and eventually omitted from our pooled estimates.

Fifth, the included patients were reasonably heterogeneous in terms of the clinical stage of the background RSV infection at the time of the diagnosis. Later diagnoses may have lost milder features (e.g., fever > 37.8 °C, cough, wheezing) or transitory ones (e.g., anomalies of eye movement) or may have inaccurately accounted for distinctive neurological features within the umbrella term of altered state of consciousness (e.g., blurred speaking, disorientation). This is even more relevant when dealing with CT, MRI, and EEG studies. Not only were imaging techniques not consistently performed for all patients but also the choice of imaging study was most likely based on local availability, eventually biasing our pooled results. Moreover, a common feature of all viral encephalitis is the progressive, diachronic involvement of distinctive areas within the CNS [63,65,183,184]: having performed specific studies in an earlier rather than a later stage of the clinical syndrome may lead to recording falsely negative cases or, conversely, associating with RSV infection more a severe pattern due to the oversampling of cases in an advanced and more complicated stage of the disease. As previously stressed, this could be of particular significance when dealing with uncommon and later complications of RSV cases [175,176,177,178,179]. In fact, due to the virology of RSV and the progressive, non-synchronous involvement of the respiratory system during the clinical infection, even RT-qPCR performed on respiratory samples could fail to identify viral RNA in a timely manner. As RT-qPCR is far from a true diagnostic “gold standard” for RSV, also estimates of Se, Sp, PPV, and NPV for CT, MRI, and EEG studies should be only cautiously acknowledged.

Finally, RSV infection is not limited to pediatric age, but the highest incidence rates are usually identified in individuals aged less than two years [1,2,14,166,185,186]. In our study, around half of the sampled case reports occurred in individuals aged less than one year: in this population, variability and characteristics of encephalopathies and symptoms such as consciousness, gait abnormalities, and speech changes might have biased (or even underestimated) the true extent of the impact of RSV on determining encephalitis. Similarly, the association of seizures with underlying encephalopathies in infants <6 months old could be overestimated given the particularly high risk for developing seizures following viral, febrile infections in such very vulnerable groups of young infants.

## 5. Conclusions

Our results suggest that RSV infection may represent a not frequent but notable cause of encephalitis cases. Even though the neuroinvasive potential of RSV was documented, most cases of encephalitis/encephalopathy associated with RSV are likely due to respiratory issues and hypoxia following LRTI. The available evidence is limited, but RSV cases were seemingly deprived of distinctive features in both clinical and radiological studies and also in EEG studies. Collectively, our data stress the importance of more accurate virological surveillance of cases of encephalitis aimed at properly characterizing the actual epidemiology of RSV infections in this specific subset of patients.

## Figures and Tables

**Table 1:** Summary of 15 observational studies included in the present systematic review and meta-analysis.

Article, Year	Country	Timeframe	Setting	Description
Ng et al., 2001 [81]	USA (Texas)	09/1994 to 05/1998	SC	Observational study from a large tertiary reference center; patients with bronchiolitis associated with RSV with any neurological complications.
Hanna et al., 2003 [82]	United Kingdom	11/1999 to 03/2000 11/2000 to 03/2001	SC	Retrospective observational study from a regional pediatric intensive care unit. The study included cases of RSV infections with neurological complications represented by seizures.
Kho et al., 2004 [53]	USA (Arizona)	01/1996 to 12/1999	SC	Retrospective observational study from a pediatric intensive care unit. Inclusion criteria were age 24 months or less; a clinical diagnosis of acute respiratory illness associated with RSV as the primary indication for admission. Cases of encephalopathy were considered by tracking apnea, seizure, persistent objective evidence of neurologic deficit at discharge, request for consultation by the child neurology service/neurorehabilitation service/physical or occupational or speech therapy.
Sweetman et al., 2005 [52]	USA (Arizona)	04/1999 to 02/2003	SC	Children aged less than 14 years with bronchiolitis having positive results on RSV and any neurological complication based on the discharge-coding database.
Millichap et al., 2009 [55]	USA (Illinois)	01/2006 to 01/2008	SC	Retrospective observational study from a tertiary center. Patients were included if they had a diagnosis of RSV infection and any neurological consultation.
Park et al., 2014 [83]	South Korea	01/2005 to 01/2012	SC	Retrospective analysis of clinical charts from patients admitted to the pediatric department. RSV infections were identified from RT-qPCR on biological specimens; neurological cases were identified by request of imaging studies (computer tomography or magnetic resonance imaging).
Cha et al., 2019 [84]	South Korea	01/2011 to 12/2016	SC	Retrospective analysis of medical records of patients aged 15 years or less with RSV infection confirmed by laboratory analysis, neurologic symptoms with seizures.
Beattie et al., 2013 [85]	USA (California)	1998 to 2010	MC	Retrospective analysis of cases with encephalitis with suspected involvement of thalamus and/or basal ganglia. Inclusion criteria were hospitalization ≥ 24 h with encephalopathy and one or more of the following findings: fever, seizure, focal neurologic findings, CSF pleocytosis, abnormal encephalogram, abnormal neuroimaging.
Ahn et al., 2016 [86]	South Korea	2000 to 2015	SC	Retrospective observational study on cases of meningoencephalitis; the study included cases with clinical suspicion of CNS infection, aged 18 years or more, with pleocytosis at CSF. A study with more limited timeframe (2012–2015) and 661 patients was published in 2020 from the same authors [91].
Hon et al., 2016 [87]	China (Hong Kong)	10/2002 to 12/2014	SC	Children admitted to a pediatric intensive care unit with diagnosis of encephalitis (i.e., clinical evidence or neuroimaging abnormalities).
Fowler et al., 2018 [88]	Sweden	2000 to 2004	SC	Children admitted to a large pediatric center with retrospective diagnosis of encephalitis (altered consciousness, personality or behavioral changes lasting for more than 24 h or two abnormal ECG findings plus anormal neuroimaging or positive focal neurological findings, seizures) and signs of inflammation.
Kawasaki et al., 2019 [89]	Japan	1986 to 2014	MC	Retrospective analysis of children with diagnosis of acute encephalitis or encephalitis and RSV infection. Acute encephalopathy was defined as a disorder of CNS with altered or loss of consciousness > 24 h after acute onset.
Fan et al., 2023 [69]	China (Mainland)	06/2020 to 09/2020	SC	Samples collected from children hospitalized with a previous diagnosis of encephalitis or meningitis or suspected in accordance with clinical diagnostic criteria.
Galardi et al., 2023 [80]	Myanmar	2016 to 2018	SC	Prospective recruitment of children aged <12 years with a diagnosis of acute or subacute encephalitis, defined by altered mental status lasting at least 24 h and at least two of the following findings: fever ≥ 38 °C in 72 h, new onset seizures, new onset focal neurological findings, pleocytosis, new/acute MRI brain abnormalities suggestive of encephalitis, abnormal electroencephalographic findings considered consistent with encephalitis.
Nicholson et al., 2024 [81]	USA (Texas)	04/2014 to 10/2014	SC	Nested cohort study recruiting all individuals aged 15 days to 20 years if they presented to the Emergency Department with a temperature of ≥38 °C with a history of fever and CSF collection.

Note: CNS = central nervous system; CSF = cerebrospinal fluid; SC = single center; MC = multicenter; RSV = respiratory syncytial virus.

**Table 2:** Characteristics of seven studies included in the present systematic review and meta-analysis reporting the occurrence of encephalitis/encephalopathy cases (i.e., neurological cases) on diagnoses of RSV infections.

Article, Year	Country	Timeframe	Diagnosis	Total RSV Cases (N.)	Neurological Cases (n./N., %)
Ng et al., 2001 [81]	USA (Texas)	09/1994 to 05/1998	IIF + viral cultures	487	9 (1.85%)
Hanna et al., 2003 [82]	United Kingdom	11/1999 to 03/2000 11/2000 to 03/2001	IIF + viral cultures	130	4 (3.08%)
Kho et al., 2004 [53]	USA (Arizona)	01/1996 to 12/1999	RT-qPCR	309	121 (39.2%)
Sweetman et al., 2005 [52]	USA (Arizona)	04/1999 to 02/2003	IIF + viral cultures	964	12 (1.24%)
Millichap et al., 2009 [55]	USA (Illinois)	01/2006 to 01/2008	RT-qPCR	780	9 (1.15%)
Park et al., 2014 [83]	Turkey	01/2005 to 01/2012	RT-qPCR	3856	8 (0.21%)
Cha et al., 2019 [84]	South Korea	01/2011 to 12/2016	RT-qPCR	1193	35 (2.93%)

Note: IIF = indirect immune fluorescence; RT-qPCR = real-time quantitative polymerase chain reaction; RSV = respiratory syncytial virus.

**Table 3:** Characteristics of eight studies included in the present systematic review and meta-analysis reporting the proportion of respiratory syncytial virus (RSV) cases over the whole of diagnoses of encephalopathy.

Article, Year	Country	Timeframe	Diagnosis	Total Cases (N.)	RSV Cases (n./N., %)	
					Respiratory Specimens	CSF
Beattie et al., 2013 [85]	USA (California)	1998 to 2010	RT-qPCR	183	-	2 (1.09%)
Ahn et al., 2016 [86]	South Korea	2000 to 2015	RT-qPCR	681	-	3 (0.44%)
Hon et al., 2016 [87]	China (Hong Kong)	10/2002 to 12/2014	RT-qPCR	46	2 (4.3%)	0 (-)
Fowler et al., 2018 [88]	Sweden	2000 to 2004	IIF	93	6 (6.45%)	0 (-)
Kawasaki et al., 2019 [89]	Japan	1986 to 2014	IIF	280	6 (2.14%)	0 (-)
Fan et al., 2023 [69]	China (Mainland)	06/2020 to 09/2020	RT-qPCR	179	-	0 (-)
Galardi et al., 2023 [80]	Myanmar	2016 to 2018	RT-qPCR	43	-	1 (2.33%)
Nicholson et al., 2024 [81]	USA (Texas)	04/2014 to 10/2014	RT-qPCR	126	-	0 (-)

Note. IIF = indirect immune fluorescence; RT-qPCR = real-time quantitative polymerase chain reaction; CSF = cerebrospinal fluid.

**Table 4:** Summary of clinical features of RSV-related cases of encephalopathy; data retrieved from case-crossover studies.

Article, Year	N./210 (%)	Age Range	Prematurity (n./N., %)	Age < 1 Year (n./N., %)	Males (n./N., %)	LRTI (n./N., %)	Intubation (n./N., %)	Apnea (n./N., %)	Cardiac Arrest (n./N., %)	Seizures (n./N.,%)
Ng et al., 2001 [81]	9 (4.29%)	6 w to 2 y	3 (33.33%)	5 (55.56%)	4 (44.44%)	9 (100%)	3 (33.33%)	1 (11.11%)	1 (11.11%)	9 (100%)
Hanna et al., 2003 [82]	4 (1.90%)	6 w to 12 w	2 (50.00%)	4 (100%)	2 (50.00%)	4 (100%)	3 (75.00%)	3 (75.00%)	n.a.	4 (100%)
Kho et al., 2004 [53]	121 (57.62%)	1 w to 22 m	n.a.	n.a.	87 (71.90%)	121 (100%)	38 (31.40%)	24 (19.83%)	n.a.	8 (6.61%)
Sweetman et al., 2005 [52]	12 (5.71%)	4 w to 19 m	8 (66.67%)	8 (66.67%)	6 (50.00%)	12 (100%)	n.a.	n.a.	n.a.	7 (58.33%)
Fowler et al., 2008 [88]	6 (2.86%)	1 m to 1.1 y	n.a.	n.a.	n.a.	n.a.	n.a.	n.a.	n.a.	2 (33.33%)
Millichap et al., 2009 [55]	9 (4.29%)	5 w to 3 y	5 (55.56%)	8 (88.89%)	5 (55.56%)	9 (100%)	7 (77.78%)	4 (44.44%)	4 (44.44%)	4 (44.44%)
Park et al., 2014 [83]	8 (3.81%)	4 m to 4 y	n.a.	3 (37.50%)	7 (87.50%)	3 (37.50%)	n.a.	n.a.	n.a.	4 (50.00%)
Cha et al., 2019 [84]	35 (16.67%)	7 w to 7.2 y	n.a.	14 (40.00%)	19 (54.29%)	27 (77.14%)	n.a.	n.a.	n.a.	35 (100%)
Kawasaki et al., 2019 [89]	6 (2.86%)	8 m to 7 y	0 (-)	1 (16.67%)	2 (33.33%)	6 (100%)	3 (50.00%)	6 (100%)	0 (-)	6 (100%)
Prev. (95%CI)	-	-	52.94% (36.46; 68.81)	57.02% (36.48; 75.40)	60.33% (48.47; 71.10)	99.55% (58.04; 100)	47.53% (27.71; 68.16)	48.27% (14.84; 83.32)	1.00% (0.02; 33.47)	83.96% (33.07; 98.23)
I^2^	-	-	0.0% (0.0; 84.7)	34.3% (0.0; 72.2)	38.9% (0.0; 73.0)	0.0% (0.0; 67.6)	55.1% (0.0; 83.4)	46.6% (0.0; 80.4)	0.0% (0.0; 74.6)	71.2% (43.2; 85.4)
Tau^2^	-	-	0.0	0.827	0.090	13.104	0.365	2.639	8.284	8.704
Q (*p* value)	-	-	2.37 (0.499)	17.93 (0.006)	12.33 (0.090)	48.52 (<0.001)	10.72 (0.030)	25.52 (<0.001)	25.92 (<0.001)	171.80 (<0.001)

Note: RSV = respiratory syncytial virus; LRTI = lower respiratory tract infection; CSF = cerebrospinal fluid; 95%CI = 95% confidence interval).

**Table 5:** Imaging and laboratory features of RSV-related cases of encephalopathy, data retrieved from case-crossover studies.

Article, Year	CT Anomalies (n./N., %)	MRI Anomalies (n./N., %)	EEG Anomalies (n./N., %)	CSF Anomalies (n./N., %)	RSV Within the CSF (n./N., %)
Ng et al., 2001 [81]	0/5 (-)	0/5 (-)	6/9 (66.67%)	0/8 (-)	0/8 (-)
Hanna et al., 2003 [82]	0/2 (-)	1/1 (100%)	n.a.	n.a.	n.a.
Kho et al., 2004 [53]	0/9 (-)	0/9 (-)	4/12 (33.33%)	12/30 (40.00%)	0/30 (-)
Sweetman et al., 2005 [52]	1/4 (25.00%)	3/4 (75.00%)	4/8 (50.00%)	0/3 (-)	0/3 (-)
Fowler et al., 2008 [88]	1/3 (33.33%)	n.a.	5/6 (83.33%)	0/3 (-)	0/3 (-)
Millichap et al., 2009 [55]	0/2 (-)	1/5 (20.00%)	7/8 (87.5%)	0/3 (-)	0/3 (-)
Park et al., 2014 [83]	n.a.	3/8 (37.50%)	n.a.	1/3 (33.33%)	n.a.
Cha et al., 2019 [84]	n.a.	4/26 (15.38%)	6/21 (28.57%)	5/21 (23.91%)	0/21 (-)
Kawasaki et al., 2019 [89]	5/6 (83.33%)	2/6 (33.33%)	5/6 (83.33%)	5/5 (100%)	1/6 (16.67%)
Prev. (95%CI)	9.23% (0.73; 58.48)	20.62% (12.33; 32.41)	60.21% (38.80; 78.31)	12.88% (1.32; 62.04)	0.60% (0.01; 48.13)
I^2^	0.0% (0.0; 70.8)	0.0% (0.0; 67.6%)	55.5% (0.0; 80.9)	0.0% (0.0; 67.6)	0.0% (0.0; 67.6)
Tau^2^	4.933	0.013	0.725	6.240	2.558
Q (*p* value)	19.39 (0.004)	13.01 (0.072)	17.02 (0.009)	28.05 (<0.001)	5.72 (0.573)

Note: RSV = respiratory syncytial virus; CT = computed tomography; MRI = magnetic resonance imaging; EEG = electroencephalogram; CSF = cerebrospinal fluid.

**Table 6:** Clinical features of 84 cases of respiratory syncytial virus (RSV) related encephalopathy/encephalitis.

Variable	N./84 (%)	RSV Documented within the Brain and/or CSF		*p* Value ^a^
		Yes (N./15, %)	No (N./69, %)	
Demographics				
Age (years, average ± SD)	4.4 (11.7)	4.3 (6.0)	4.4 (12.5)	0.051 ^b^
Age < 1 year	39 (46.43%)	4 (26.67%)	35 (50.72%)	0.159
Prematurity	8 (9.52%)	1 (6.67%)	7 (10.14%)	1.000
Male gender	47 (55.95%)	11 (73.33%)	36 (52.17%)	0.227
Presenting symptoms				
Fever (temperature > 37.8 °C)	41(48.81%)	10 (66.67%)	31 (44.93%)	0.214
Cough	32 (38.10%)	5 (33.33%)	27 (39.13%)	0.900
Wheezing	10 (11.90%)	0 (-)	10 (14.49%)	0.258
Ataxia	11 (13.10%)	3 (20.00%)	8 (11.59%)	0.651
Dyspnea/tachypnea	22 (26.19%)	4 (26.67%)	18 (26.09%)	1.000
Altered consciousness	36 (42.86%)	11 (73.33%)	25 (36.23%)	0.019
Disorientation	12 (14.29%)	4 (26.67%)	8 (11.59%)	0.269
Aphasia/slurred speech	6 (7.14%)	2 (13.33%)	3 (4.35%)	0.465
Anomalies of eye movement	13 (15.48%)	2 (13.33%)	11 (15.94%)	1.000
Seizures	55 (65.48%)	11 (73.33%)	44 (63.77%)	0.684
Apnea	10 (11.90%)	2 (13.33%)	8 (11.59%)	1.000
Clinical features compatible with…				
Influenza-like illness	23 (27.38%)	8 (53.33%)	15 (21.74%)	0.030
Lower respiratory tract illness	12 (14.29%)	3 (20.00%)	9 (13.04%)	0.771
RSV detected within the brain/CSF	15 (17.86%)	-	-	-
Outcome				
Cardiac arrest	11 (13.10%)	4 (26.67%)	7 (10.14%)	0.195
Intubation	23 (27.38%)	2 (13.33%)	21 (30.43%)	0.305
ECMO	2 (2.38%)	0 (-)	2 (2.90%)	1.000
Status				0.081
Recovery (full)	29 (34.52%)	4 (26.67%)	25 (36.23%)	
Recovery (partial)	19 (22.62%)	8 (53.33%)	11 (15.94%)	
Death	9 (10.71%)	3 (20.00%)	6 (8.70%)	
Undisclosed	27 (32.14%)	0 (-)	27 (39.13%)	

Note = (^a^) if not otherwise specified, chi-squared test with continuity correction; (^b^) Mann–Whitney U test *p* value; SD = standard deviation; CSF = cerebrospinal fluid.

**Table 7:** Features of 84 cases of respiratory syncytial virus (RSV)-related encephalopathy associated with RSV findings in the central nervous system (either cerebrospinal fluid or brain tissue). Association was assessed by calculation of adjusted odds ratio with their 95% confidence intervals through binary logistic regression analysis.

Variable	Adjusted Odds Ratio (95% Confidence Interval)
Male gender	5.021 (1.104; 22.831)
<1 year of age	0.717 (0.142; 3.621)
Fever	0.907 (0.186; 4.422)
Altered consciousness	2.080 (0.440; 9.823)
Clinical features compatible with ILI	2.420 (0.557; 10.516)
Cardiac arrest	2.158 (0.177; 26.249)
Outcome	
Full recovery	1.000 (reference)
Partial recovery	5.699 (1.152; 28.183)
Death	2.052 (0.128; 32.963)

Note: ILI = influenza-like illness.

**Table 8:** Diagnostic performances of computed tomography, magnetic resonance imaging, and electroencephalogram for identification of respiratory syncytial virus infections within the central nervous system (either cerebrospinal fluid or brain tissue).

Diagnostic Procedure	No. of Exams (No./84, %)	Any Finding (n/No., %)	Se. (95%CI)	Sp. (95%CI)	PPV (95%CI)	PNV (95%CI)
Computed tomography	46 (54.76%)	22 (47.83%)	0.455 (0.212; 0.720)	0.514 (0.356; 0.670)	0.227 (0.101; 0.434)	0.750 (0.551; 0.880)
Magnetic resonance imaging	56 (66.67%)	41 (73.21%)	0.900 (0.596; 0.995)	0.304 (0.191; 0.448)	0.220 (0.120; 0.367)	0.933 (0.702; 0.997)
Electroencephalogram	39 (46.43%)	31 (79.49%)	1.000 (0.610; 1.000)	0.242 (0.128; 0.430)	0.194 (0.092; 0.363)	1.000 (0.676; 1.000)

Note: Se. = sensitivity; Sp. = specificity; PPV = predictive positive value; PNV = predictive negative value.

**Table 9:** Summary of characteristics of 56 cases of encephalopathy associated with respiratory syncytial virus infection obtained from magnetic resonance imaging.

Main Affected Site at Magnetic Resonance Imaging	No./56 (%)	Respiratory Syncytial Virus Documented within the Brain and/or Cerebrospinal Fluid		*p* Value
		Yes (No./10, %)	No (No./46, %)	
Not reported	17 (30.36%)	2 (20.0%)	17 (36.96%)	0.511
Diffuse damage	23 (41.07%)	4 (40.0%)	19 (41.30%)	1.000
Neocortex (in general)	13 (23.21%)	4 (40.0%)	9 (19.57%)	0.330
Frontal cortex	7 (12.50%)	4 (40.0%)	3 (6.52%)	0.018
Temporal cortex	6 (10.71%)	3 (30.0%)	3 (6.52%)	0.107
Occipital cortex	3 (5.36%)	0 (-)	3 (6.52%)	0.956
Cerebellum	9 (16.07%)	3 (30.0%)	6 (13.04%)	0.396
Basal ganglia	8 (14.29%)	1 (10.0%)	7 (15.22%)	1.000
Corpus callosum	4 (7.14%)	0 (-)	4 (8.70%)	0.772
Mesencephalon	5 (8.93%)	1 (10.0%)	4 (8.70%)	1.000

**Table 10:** Summary of characteristics of 39 cases of encephalopathy associated with respiratory syncytial virus infection obtained from electroencephalographic (EEG) studies.

EEG Results	No./39 (%)	Respiratory Syncytial Virus Documented within the Brain and/or Cerebrospinal Fluid		*p* Value
		Yes (No./6, %)	No (No./32, %)	
Negative	8 (20.51%)	0 (-)	8 (25.00%)	0.311
Non-epileptiform abnormalities	14 (35.90%)	2 (33.33%)	12 (37.50%)	
Interictal epileptiform discharges	17 (43.59%)	4 (66.67%)	13 (40.63%)	

## Data Availability

Raw data are available on request to the chief investigator (matteo.ricco@ausl.re.it).

## Supplementary Materials

The following supporting information can be downloaded at: https://www.mdpi.com/article/10.3390/epidemiologia5030031/s1, Table S1: PRISMA checklist.

## Appendix A

**Table A1:** Research strategy and entries found by searched databases.

Database	Keywords Searched	No. of Entries Found
PubMed [MeSH]	(“Respiratory Syncytial Viruses”[Mesh] OR “Respiratory Syncytial Virus, Human”[Mesh] OR “Respiratory Syncytial Virus Infections”[Mesh]) AND (“Encephalomyelitis, Acute Disseminated”[Mesh] OR “Infectious Encephalitis”[Mesh] OR “Encephalitis”[Mesh] OR “Encephalitis, Viral”[Mesh] OR “Encephalitis Viruses”[Mesh] OR “Meningitis”[Mesh] OR “Meningitis, Viral”[Mesh])	74
SCOPUS	(“Respiratory Syncytial Viruses” OR “Respiratory Syncytial Virus, Human” OR “Respiratory Syncytial Virus Infections”) AND ((encephalomyelitis AND acute AND disseminated) OR (infectious AND encephalitis) OR encephalitis OR (encephalitis AND viral) OR (encephalitis AND virus) OR meningitis OR (virus AND meningitis) OR (viral AND meningitis))	541
EMBASE	(‘human respiratory syncytial virus’/exp OR ‘human respiratory syncytial virus’ OR ‘bronchiolitis’) AND (‘encephalitis’ OR ‘virus encephalitis’ OR ‘epidemic encephalitis’ OR ‘meningitis’ OR ‘infectious meningitis’ OR ‘virus meningitis’)	916

**Table A2:** Summary of risk of bias assessment according to the risk of bias tool from the National Toxicology Program’s Office of Health Assessment and Translation handbook [74,120] on observational studies included in the meta-analysis.

	Ng et al. [81]	Hanna et al. [82]	Kho et al. [53]	Sweetman et al. [52]	Millichap et al. [55]	Park et al. [83]	Cha et al. [84]	Bettie et al. [85]	Kawasaki et al. [89]	Nicholson et al. [90]	Fowler et al. [88]	Ahn et al. [86]	Hon et al. [87]	Galardi et al. [80]	Fan et al. [69]
Selection bias															
Did selection of study participants result in appropriate comparison groups?															
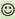
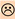
Confounding bias															
Did the study design or analysis account for important and modifying variables?															
Exclusion bias															
Were outcome data complete without attrition or exclusion from analysis?															
Detection bias															
Can we be confident in the exposure characterization?															
Can we be confident in the outcome assessment?															
Selective reporting bias															
Were all measured outcomes reported?															
Other sources of bias															
Were there no other potential threats to internal validity (e.g., statistical methods were appropriate and researchers adhered to the study protocol)?															

Note:  = definitively low;  = probably low;  = probably high;  = definitively high.

**Table A3:** Summary of the methodological quality of included cases reports and case series according to Murad et al. [72].

Study	D1	D2	D3	D4	D7	D8	Score (0 to 6)
Ahn et al. [91]							4
Al-Maskari et al. [93]							5
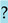
Appleberry and De Jesus [94]							4
Bo Cheng et al. [95]							6
Bottino et al. [96]							6
Erdogan et al. [97]							6
Giacchetti et al. [98]							4
Hirayama et al. [99]							6
Kakimoto et al. [100]							5
Kawashima et al. [101]							5
Kaya et al. [102]							4
Karlik et al. [103]							6
Li et al. [104]							5
Miyamoto et al.							6
Moriyama et al. [106]							6
Nakamura et al. [107]							4
Ong et al. [108]							5
Otake et al. [109]							6
Picone et al. [110]							5
Santos et al. [111]							4
Sato et al. [112]							6
Schattner et al. [113]							2
Sugimoto et al. [114]							6
Tison—Chamberlain et al. [115]							5
Xu et al. [116]							3
Yu et al. [117]							4
Zlateva et al. [118]							4

Note: D1: “Does the patient(s) represent(s) the whole experience of the investigator (centre) or is the selection method unclear to the extent that other patients with similar presentation may not have been reported? D2: “Was the exposure adequately ascertained?”; D3: “Was the outcome adequately ascertained?”; D4: “Were other alternative causes that may explain the observation ruled out?”; D7: “Was follow-up long enough for outcomes to occur?”; D8: “Is the case(s) described with sufficient details to allow other investigators to replicate the research or to allow practitioners make inferences related to their own practice?”. Items D5 and D6 were removed as mostly relevant for the analysis of adverse drug events.  = low risk;  = unclear risk;  = high risk.
